# Supplementary material for: Surface oxidation/spin state determines oxygen evolution reaction activity of cobalt-based catalysts in acidic environment
Source: Nat Commun. 2024 Apr 9;15:3067. doi: 10.1038/s41467-024-47409-y (PMC11003995; doi:10.1038/s41467-024-47409-y)
Supplement: Supplementary file 1 — Supplementary Information [file 41467_2024_47409_MOESM1_ESM.pdf]

Supplementary information for

Surface oxidation/spin state determines oxygen evolution reaction activity of cobalt-based catalysts in acidic environment

Jinzhen Huang,*[1] Camelia Nicoleta Borca,[2] Thomas Huthwelker,[2] Nur Sena Yüzbasi,[3] Dominika Baster,[1] Mario El Kazzi,[1] Christof W. Schneider,[4] Thomas J. Schmidt [1,5] and Emiliana Fabbri*[1]

1 Electrochemistry Laboratory, Paul Scherrer Institute, CH-5232 Villigen PSI, Switzerland
E-mail: emiliana.fabbri@psi.ch; jinzhen.huang@psi.ch

2 Photon Science Division, Paul Scherrer Institute, CH-5232 Villigen PSI, Switzerland

3 Laboratory for High Performance Ceramics, Empa - Swiss Federal Laboratories for Materials Science and Technology, CH-8600 Dübendorf, Switzerland

4 Laboratory for Multiscale Materials Experiments, Paul Scherrer Institute, CH-5232 Villigen PSI, Switzerland

5 Institute for Molecular Physical Science, ETH Zurich, 8093 Zurich, Switzerland

Figures

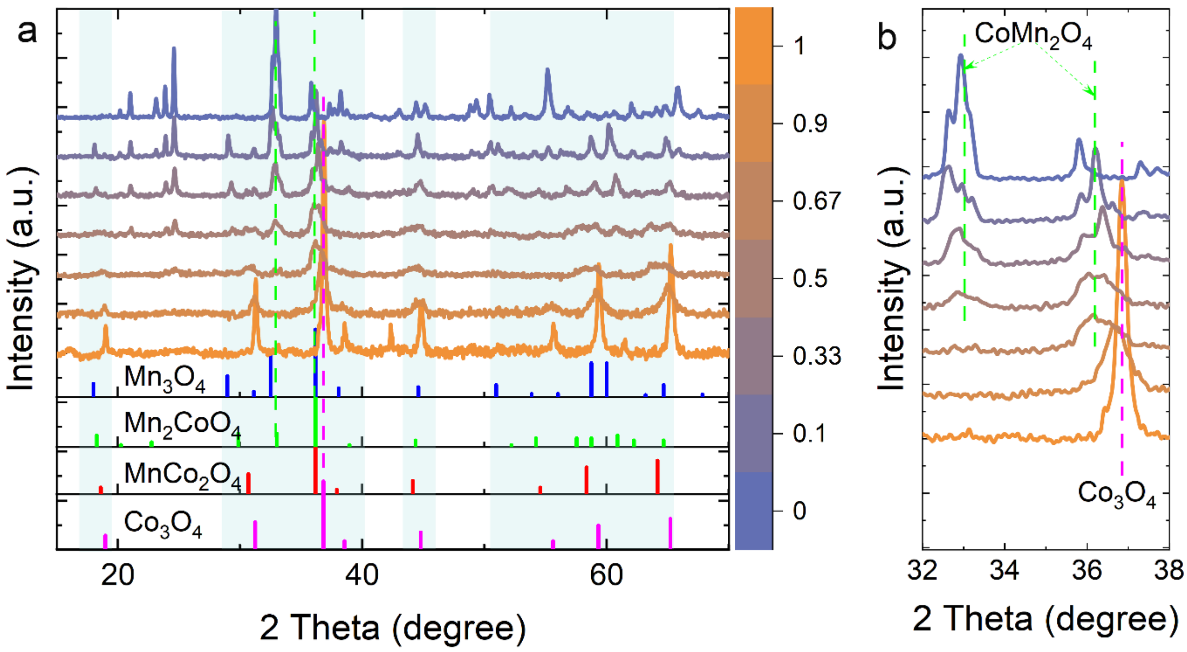

Supplementary Figure 1. (a) XRD patterns of the $Co_xMn_{1-x}O_y$ (x = 1, 0.9, 0.67, 0.5, 0.33, 0.1 and 0) catalysts. In addition to the spinel structure, there are smaller amounts of secondary phases of CoO (for x = 1) and MnO_2/Mn_2O_3 (for x < 0.9) in the samples. (b) The enlarged patterns in the range of 32 to 38 degree to show the transition from Co_3O_4 structure to $CoMn_2O_4$ structure as the Co content decreases.

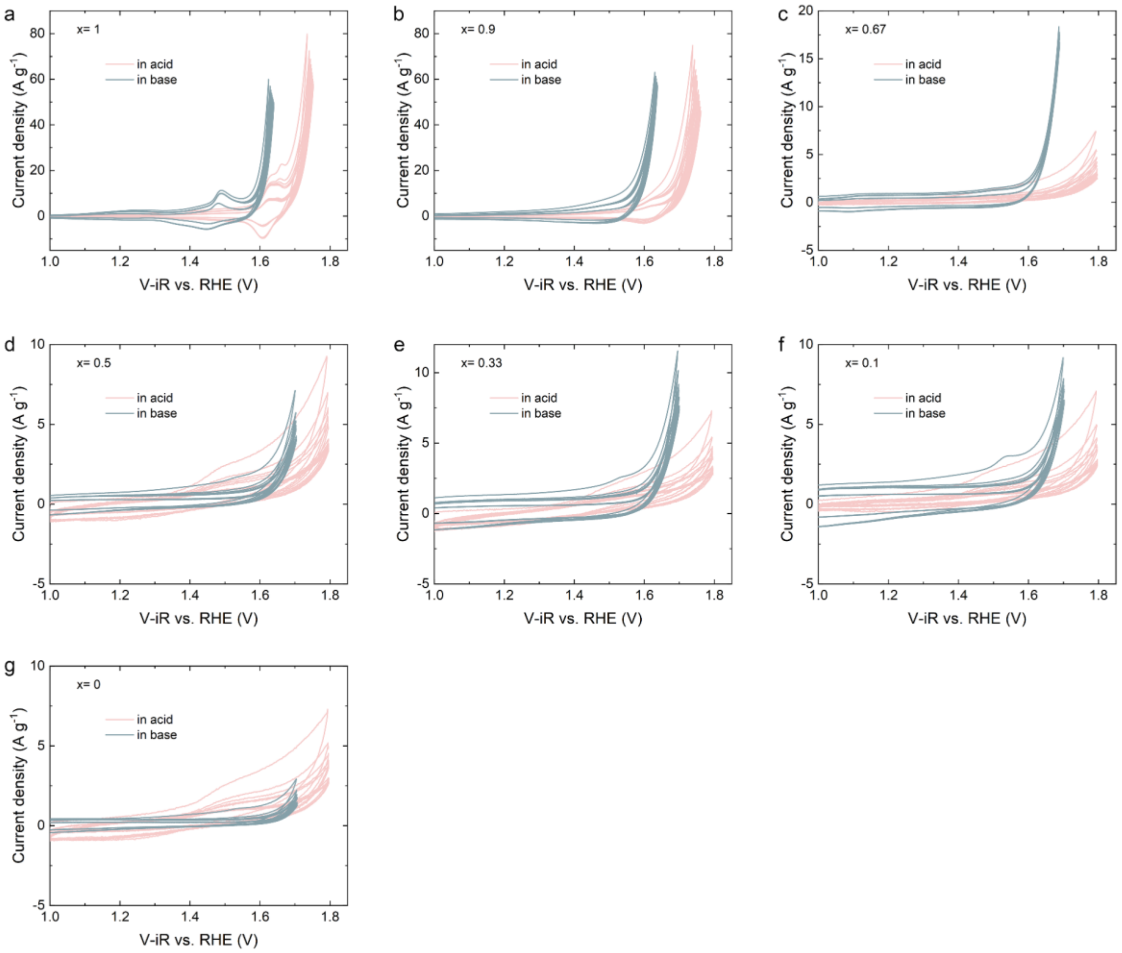

Supplementary Figure 2. The 10 CVs $Co_xMn_{1-x}O_y$ catalysts in both acidic and alkaline environments, which were collected prior to the chronoamperometric measurements. (a) x = 1, (b) x = 0.9, (c) x = 0.67, (d) x = 0.5, (e) x = 0.33, (f) x = 0.1 and (g) x = 0.

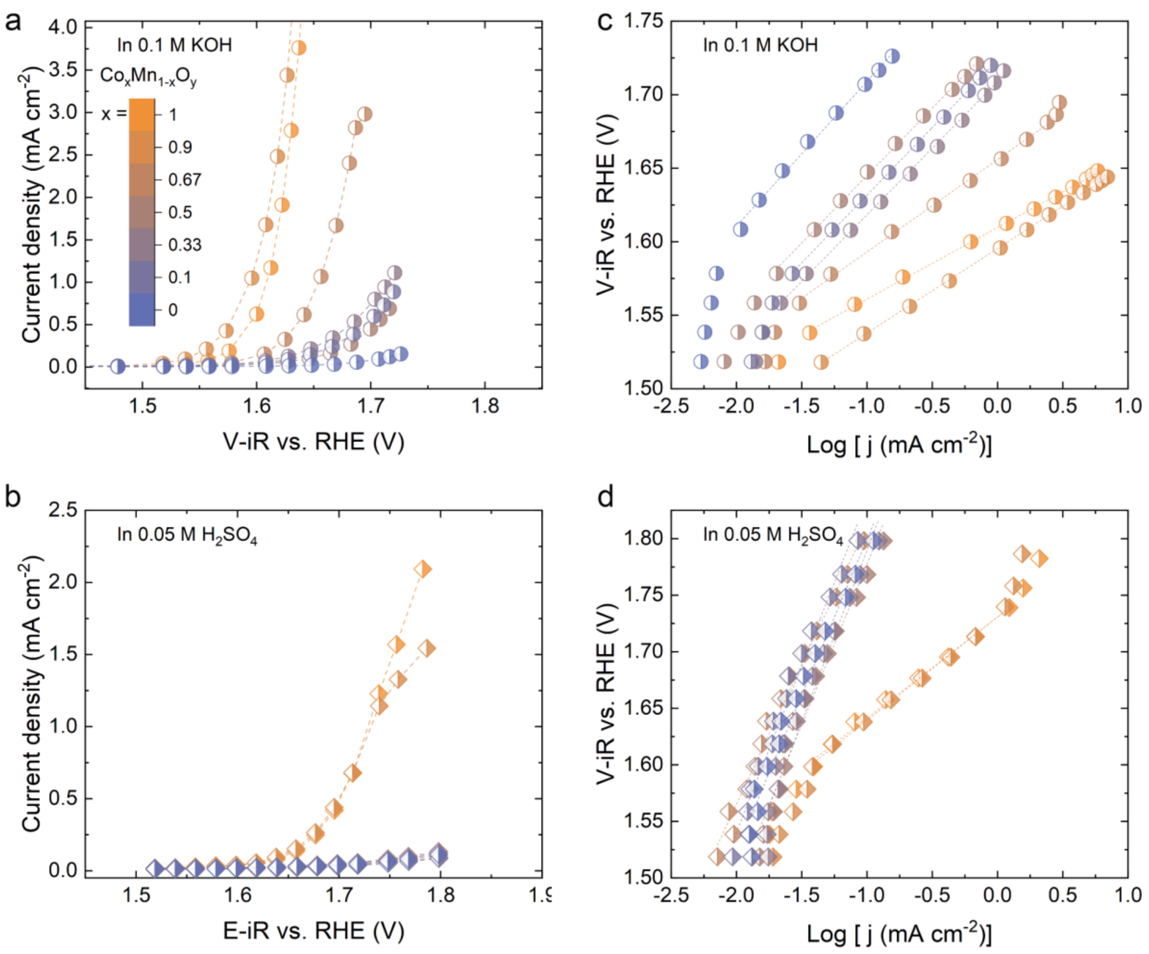

Supplementary Figure 3. Comparison of the OER activity of $Co_xMn_{1-x}\ O_y$ (x = 1, 0.9, 0.67, 0.5, 0.33, 0.1 and 0) in (a) alkaline and (b) acidic environments. (c-d) The corresponding Tafel plots. The current is normalized by the geometric area of electrode, in comparison to the results normalized by the catalyst mass loading in Fig. 1.

For each measurement, 0.02 mg of the catalyst was drop-cast on a glassy carbon disk with the surface area of 0.19625 cm^{-2}, to form a thin catalyst layer (see Method for more details). The Tafel slope, used as the major parameter for comparison in this project, would not be affected by the normalization method, since

$$0.02\ mg \times J(A\ g^{-1}) = \ 0.19625\ cm^{-2} \times J(mA\ cm^{-2})$$

Then

$$Log\ [J(A\ g^{-1})] = Log[J(mA\ cm^{-2})] + \ Log(9.8125)$$

$$\frac{\partial\ Log\ [J(A\ g^{-1})]}{\partial\ V} = \frac{\partial\ Log\ [J(mA\ cm^{-2})]}{\partial\ V}$$

$$Tafel\ slope = \frac{\partial\ V}{\partial\ Log\ [J(A\ g^{-1})]} = \frac{\partial\ V}{\partial\ Log\ [J(mA\ cm^{-2})]}$$

where V is the potential. Therefore, the different normalization methods only cause the Tafel plot shift parallel along the x-axis but would not change the Tafel slope from the fit.

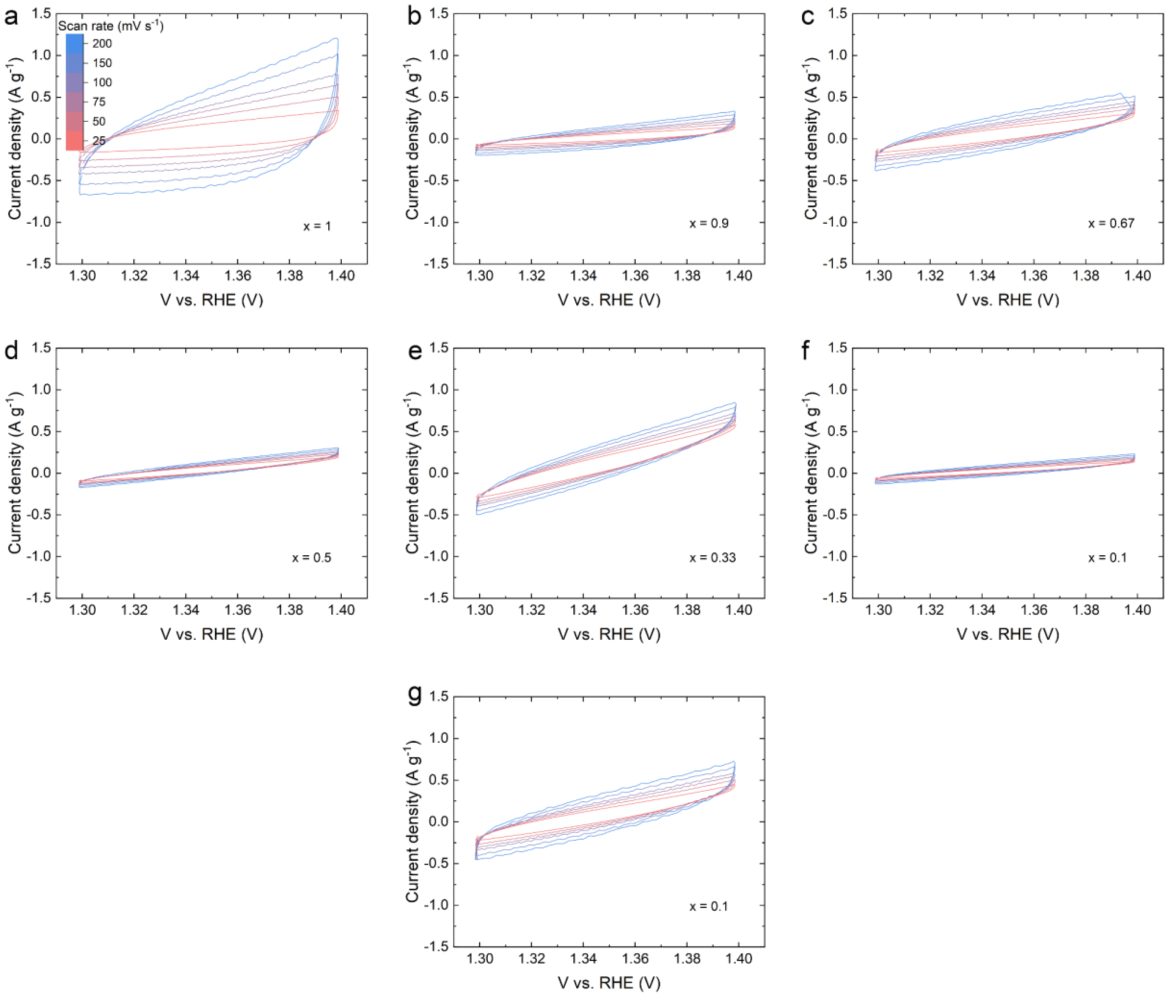

Supplementary Figure 4. (a-g) The CV profiles collected at difference scan rate (200, 150, 100, 75, 50 and 25 mV s^{-1}) for the $Co_xMn_{1-x}O_y$ samples (x = 1, 0.9, 0.67, 0.5, 0.33, 0.1 and 0). The potential window is from 1.30 to 1.40 V vs. RHE.

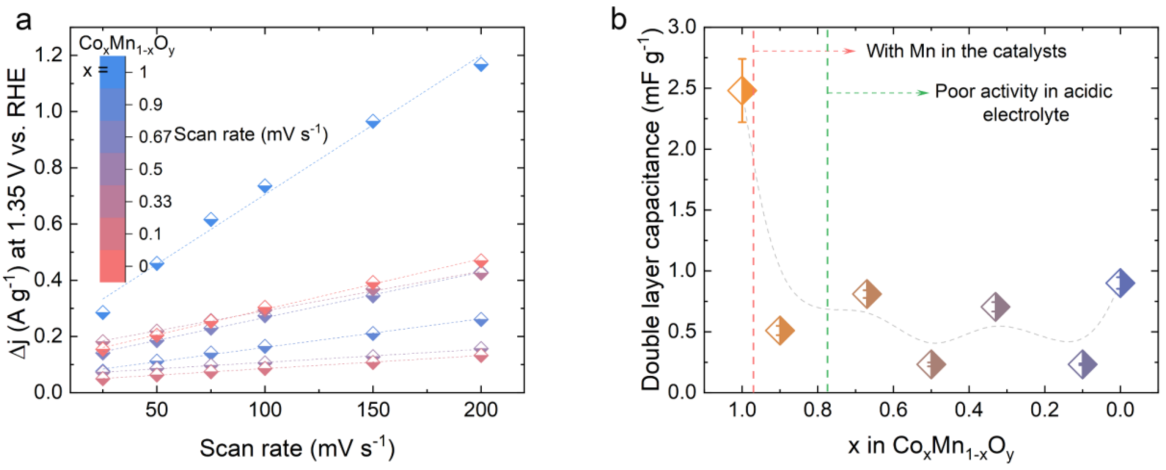

Supplementary Figure 5. (a) The current density difference (Δj) at 1.35 V vs. RHE extracted from the CVs in Supplementary Figure 4, is plotted as a function of the scan rates. (b) The extracted C_{dl} is plotted as a function of x in the $Co_xMn_{1-x}O_y$ catalysts. The error bars are from fitting.

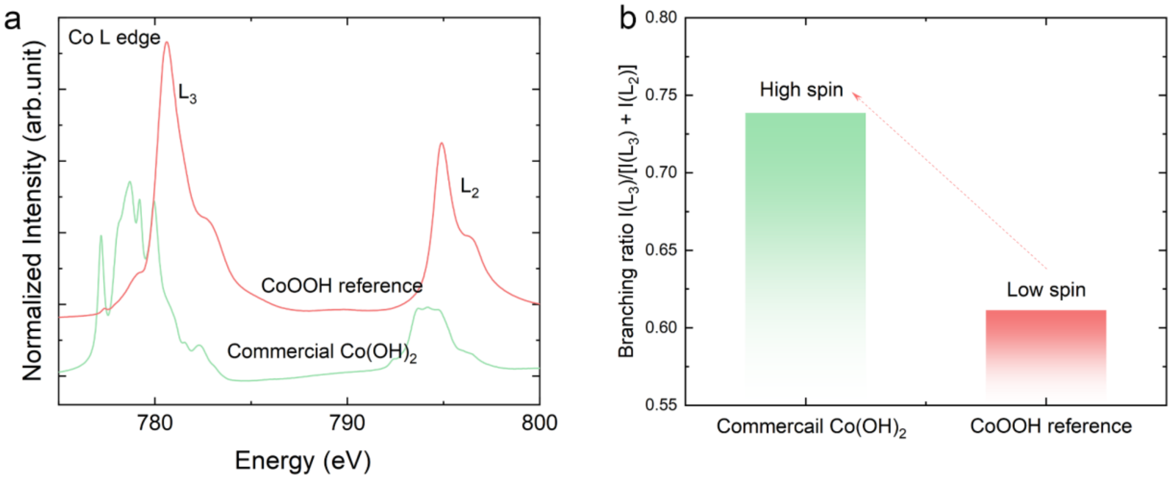

Supplementary Figure 6. Standard samples with Co atoms in the high and low spin states, respectively. (a) The Co L edge spectra of commercial $Co(OH)_2$ and CoOOH reference. (b) The calculated branching ratios.

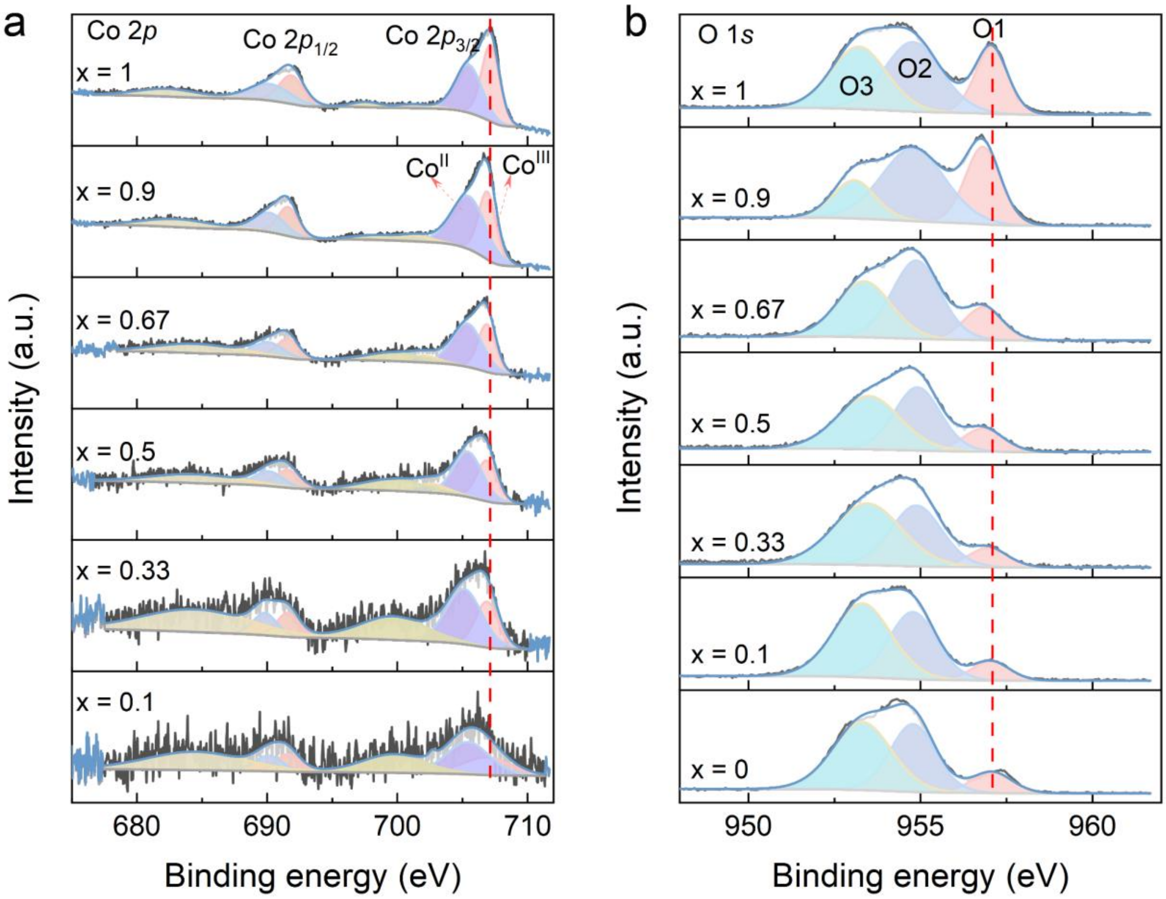

Supplementary Figure 7. XPS characterization of the $Co_xMn_{1-x}O_y$ samples at the (a) Co 2*p* and (b) O 1*s* spectra.

The main peaks of Co 2$p_{3/2}$ signal can de deconvoluted into Co^{III} and Co^{II} at ~779.6 eV and 781.4 eV respectively[1]. The Co^{II} has higher binding energy than Co^{III}, therefore the peak position of Co 2$p_{3/2}$ is shift to higher binding energy as x decreases, indicating there are more Co^{II} on the surface. The O 1*s* spectrum can be deconvoluted into three oxygen species, namely O1 is the O in the lattice, O2 is the undercoordinated oxygen defect, and O3 represent the OH^-/H_2O adsorbed on catalyst[1].

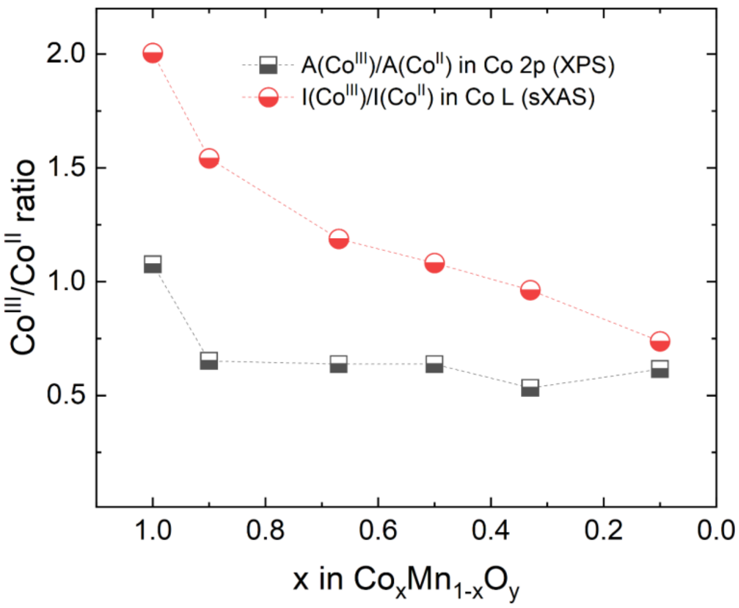

Supplementary Figure 8. Comparison of the area ratio of A(Co^{III})/A(Co^{II}) at the Co 2*p* (XPS) spectra and intensity ratio of I(Co^{III})/I(Co^{II}) at the Co L edge spectra (sXAS), respectively.

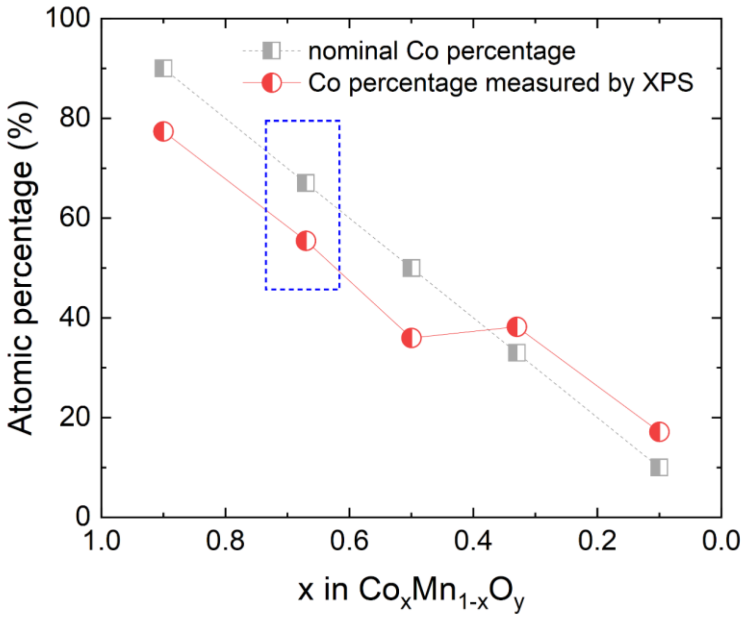

Supplementary Figure 9. The comparison of nominal Co atomic percentage used for synthesis and the Co atomic percentage measured by XPS. The Co and Mn atomic percentages measured by XPS are also summarized in Supplementary table 5.

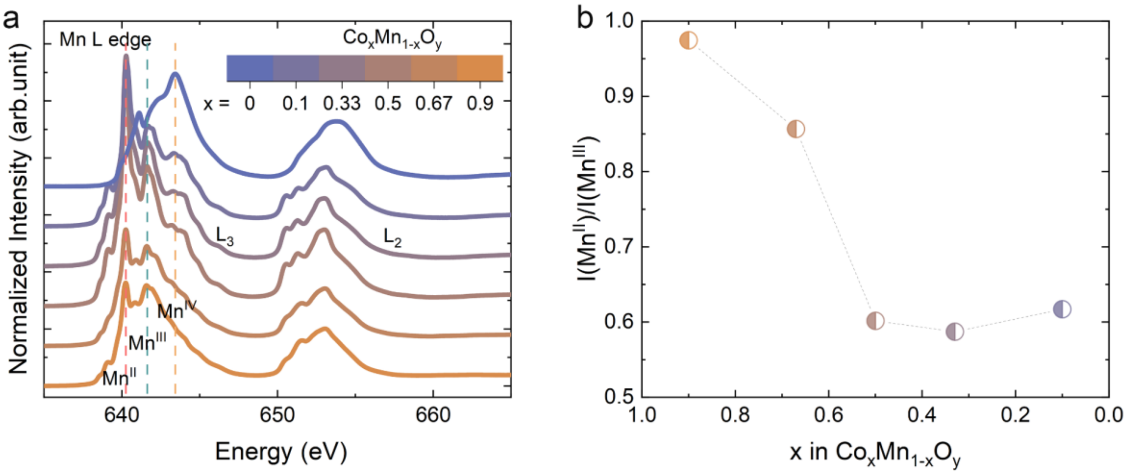

Supplementary Figure 10. (a)The Mn L edge of $Co_xMn_{1-x}O_y$ (x = 0.9, 0.67, 0.5, 0.33, 0.1 and 0). (b) The intensity ratio of $I(Mn^{II})/I(Mn^{III})$ as a function of x.

The Mn L-edge spectra show two features related to the L_3 and L_2 edge at ~ 642 and ~ 653 eV, respectively. The Mn L_3 edge features in samples with x = 0.9 and 0.67 are close to the oxidized MnO, with the white line at ~ 640.3 eV related to Mn^{II}, and another feature at ~ 641.7 eV attributed to Mn^{III} (Supplementary Fig. 10a).[2, 3] Further decreasing the Co content (x = 0.5, 0.33 and 0.1) leads to a significant increase in the Mn^{II} peak, as indicated by the higher peak intensity ratio of $I(Mn^{II})/I(Mn^{III})$ (Supplementary Fig. 10b). In addition, when there is no Co in the sample (x = 0), the white line in the Mn L_3 edge is shifted to ~ 643.5 eV, similar to that of the $Mn^{IV}O_2$.[2, 3] These observations suggest that there are strong interactions between Co and Mn at the surface.

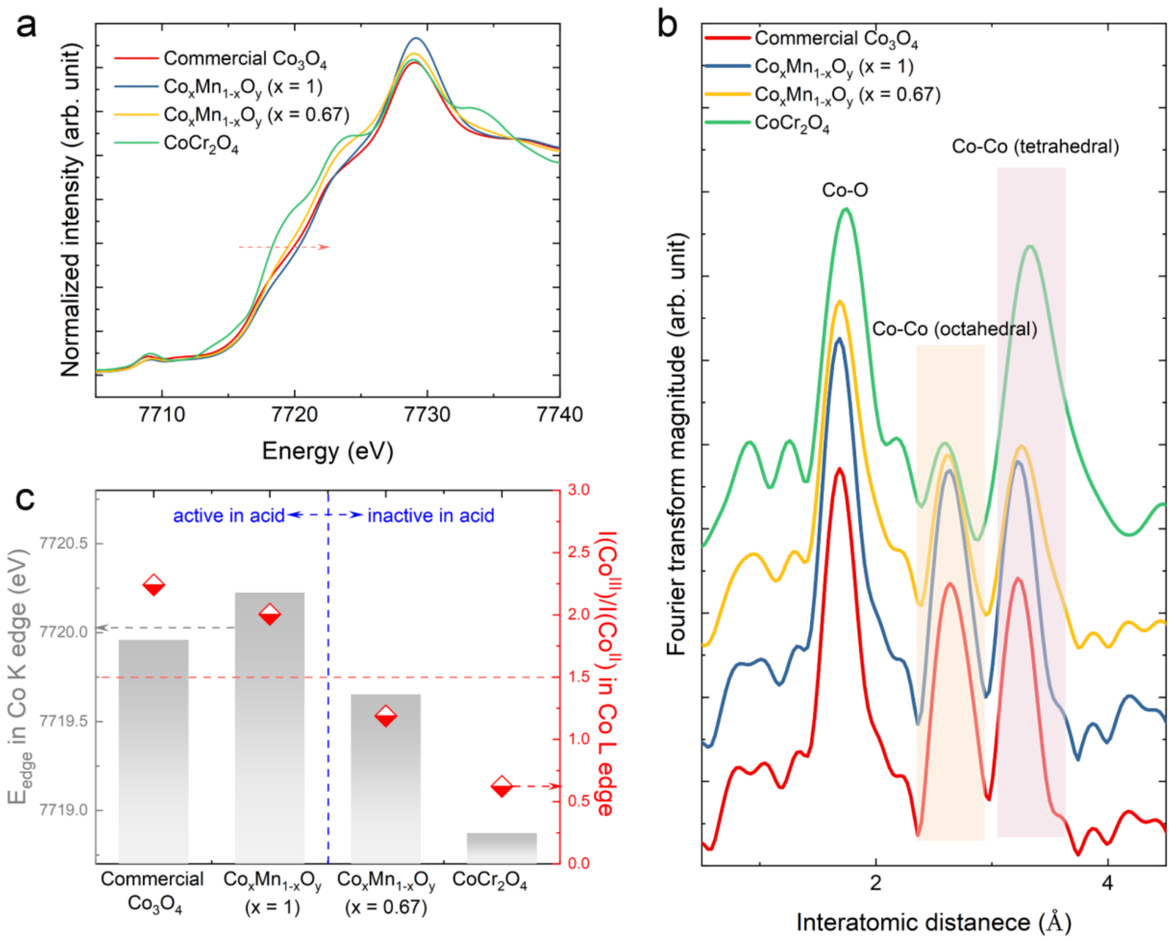

Supplementary Figure 11. Hard XAS characterization of Co K edge for four representative Co-based spinel oxides. (a) The XANES spectra, and (b) the corresponding Fourier transform EXAFS spectra. (c) The extracted Co K edge position was compared with the $I(Co^{III})/I(Co^{II})$ in Co L edge spectra.

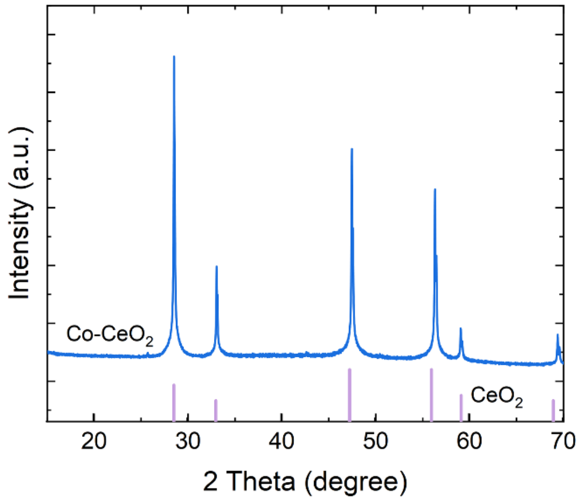

Supplementary Figure 12. The XRD pattern of Co-doped CeO_2 (Co-CeO_2), which was prepared with 5 mol% of Co and 95 mol% of Ce (in molar) by flame spray pyrolysis.

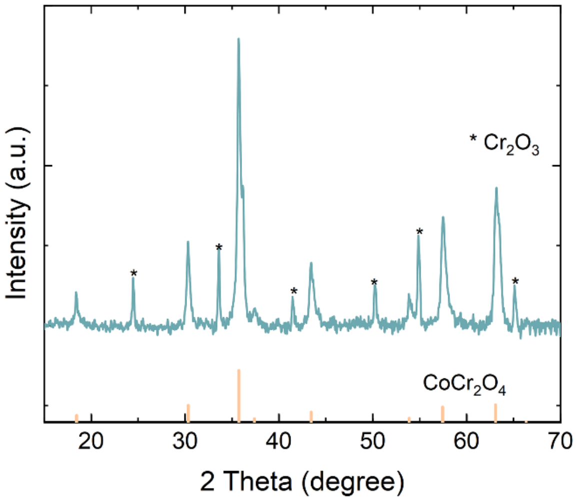

Supplementary Figure 13. The XRD pattern of $CoCr_2O_4$, which was prepared by the flame spray pyrolysis followed by annealing at 500 °C for 4 hours. The secondary phase is Cr_2O_3, which is not active towards OER. The $CoCr_2O_4$ is the normal spinel oxide, the presence of Cr_2O_3 also indicates the Cr is in excess, so the Co is occupied in the tetrahedral coordination with the oxidation state of 2+.

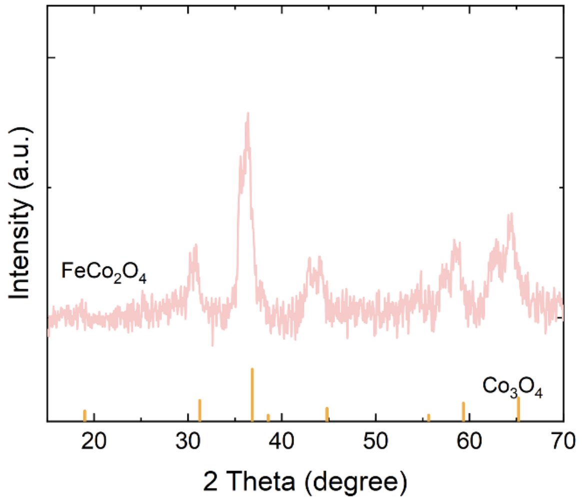

Supplementary Figure 14. The XRD pattern of $FeCo_2O_4$, which is prepared by the flame spray pyrolysis followed by annealing at 500 °C for 4h.

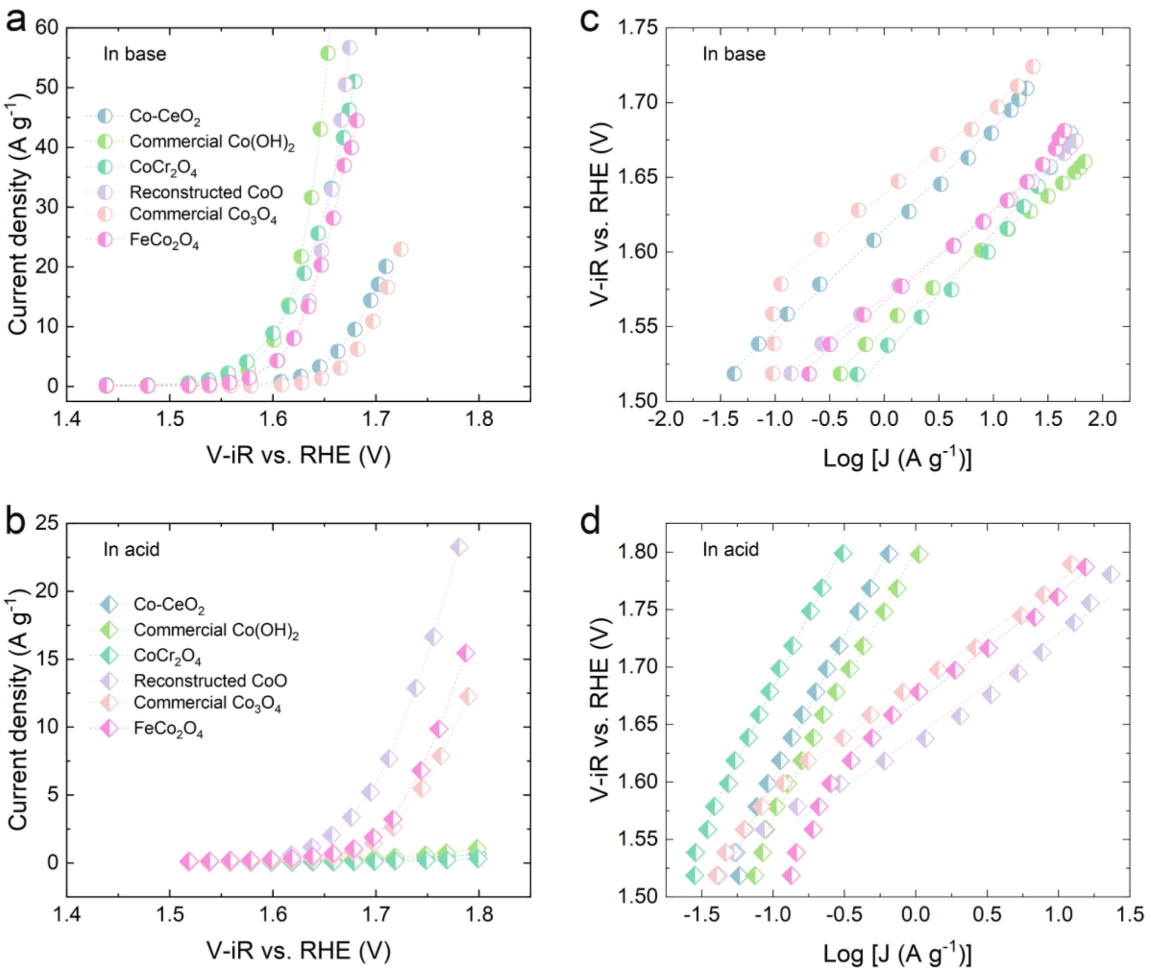

Supplementary Figure 15. The OER activity of the six representative samples with/without low-spin Co^{III} at the surface, in both alkaline and acidic environments. The polarization curves obtained by choroamperometry (a) in an alkaline environment and (b) in an acidic environment. (c-d) The corresponding Tafel plots that are derived from the polarization curves.

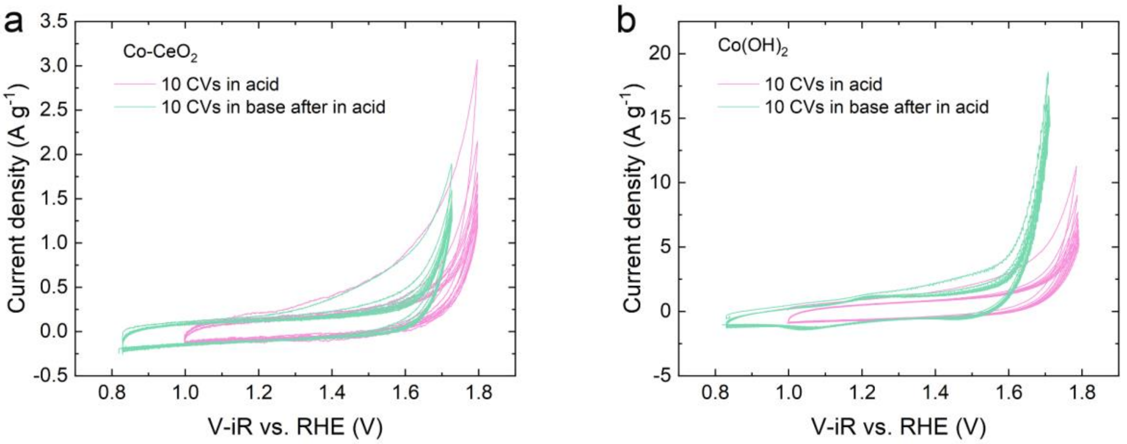

Supplementary Figure 16. The CVs (100 mV s^{-1}) for (a) Co-CeO$_2$ and (b) commercial Co(OH)$_2$ in both base (0.1 M KOH) and acid (0.05 M H_2SO_4). The electrode with catalyst was first subjected to 10 cycles of CV in an acidic environment, and then the same electrode was transferred in an alkaline environment to run another 10 cycles of CVs. Both samples suffer from Co dissolution, but the OER performance in an alkaline environment is still better in an acidic environment, so Co is not fully dissolved during the CV cycles in an acidic environment.

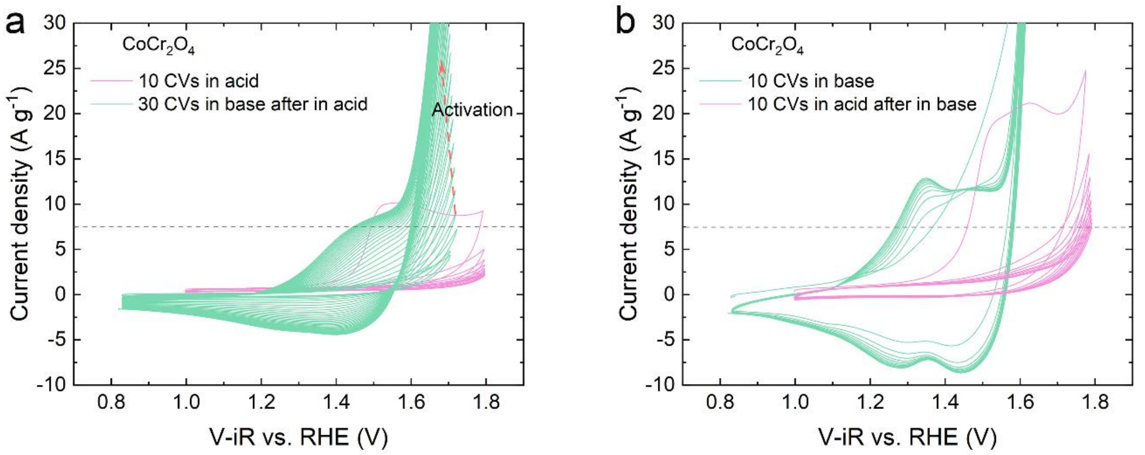

Supplementary Figure 17. The CVs (100 mV s^{-1}) of $CoCr_2O_4$ in both base and acid but with different protocols. (a) The $CoCr_2O_4$ was first subjected to run 10 CVs in an acidic environment, and then it was transferred to an alkaline environment for another 30 cycles. During the latter process, the catalyst can be re-activated again. (b) The $CoCr_2O_4$ was first subjected to run 10 CVs in an alkaline environment, with slight activation, and then it was transferred to an acidic environment for further 10 cycles.

The $CoCr_2O_4$ catalyst is inactive in acid, but it can still be activated in an alkaline environment, proving that the poor OER activity in acid is not due to complete dissolution of the catalyst. Furthermore, when $CoCr_2O_4$ was first activated in an alkaline environment to have reconstructed oxy-hydroxide layer, the OER performance in acid is slightly improved compared to the pristine electrode, manifesting the surface reconstruction to form surface Co^{III} is not favorable in acid.

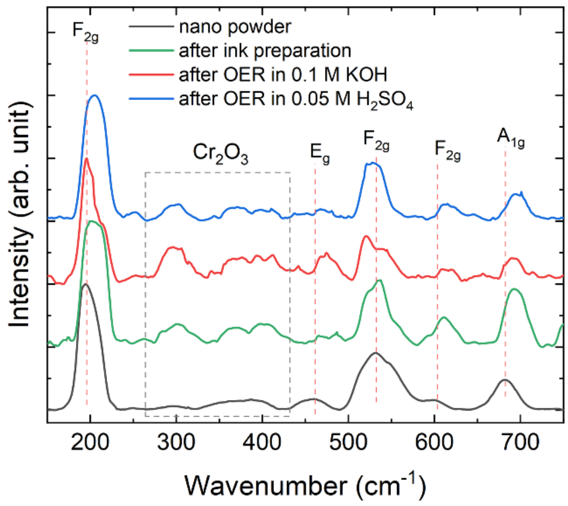

Supplementary Figure 18. Raman characterization of the pristine $CoCr_2O_4$ nano-powder and its counterparts after ink preparation, after OER in 0.1 M KOH and 0.05 M H_2SO_4, respectively.

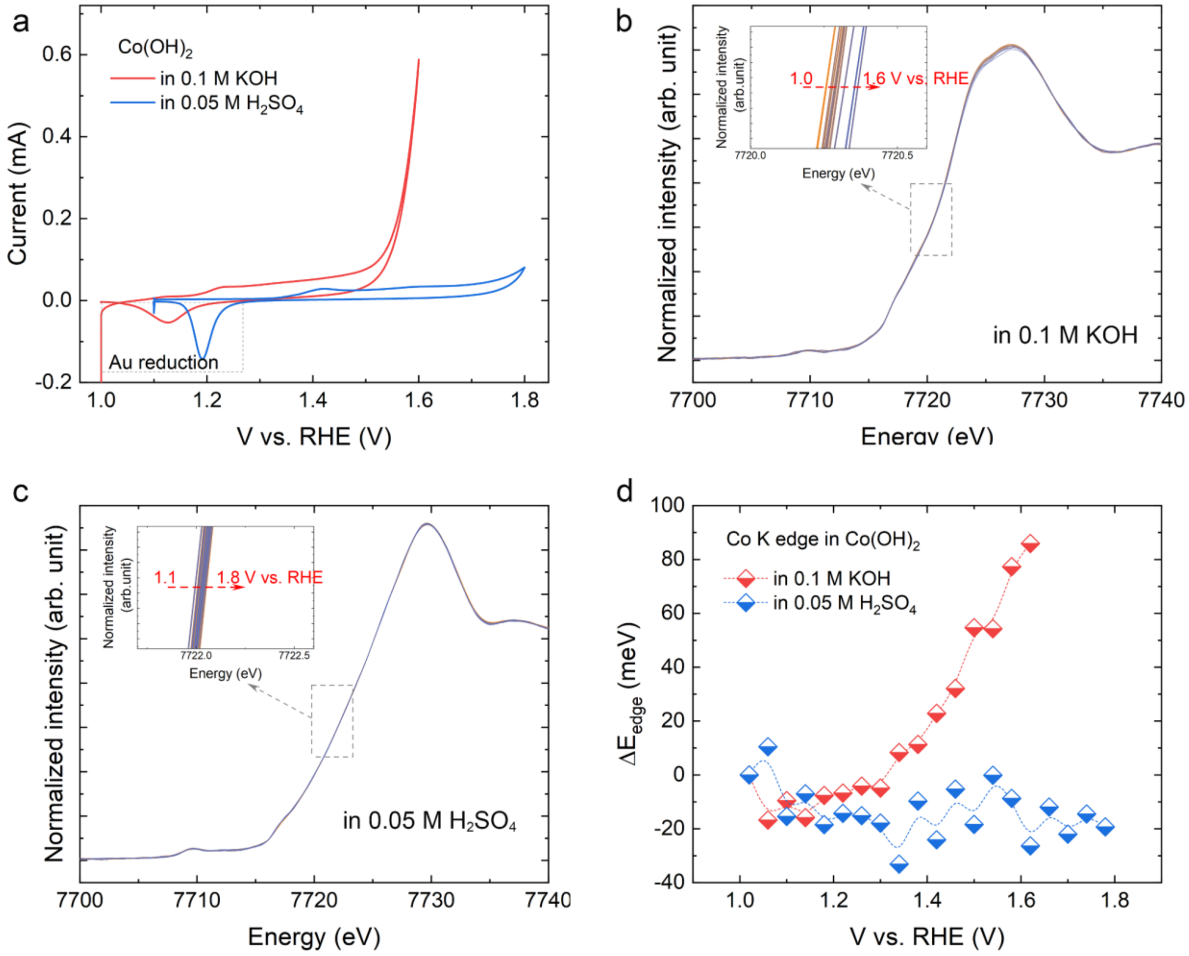

Supplementary Figure 19. Operando hard XAS characterization of Co K edge in the commercial $Co(OH)_2$ catalyst to reveal the pH-dependent Co oxidation behavior during OER. (a) The CV profiles collected in a spectro-electrochemical cell[4], with a scan rate of 4 mV s^{-1}. The operando XANES spectra of Co K edge collected in (b) 0.1 M KOH and (c) 0.05 M H_2SO_4, respectively. (d) The change of the Co K edge (ΔE_{edge}) is plotted as a function of the applied potential.

The ΔE_{edge} was obtained by following equation:

$$\Delta E_{edge} = E_{edge}(V_f) - E_{edge}(V_i)$$

where E_{edge} (V_f) is the Co K edge position extracted at different applied potential of V_f, $E_{edge}(V_i)$ is the Co K edge position at the initial potential of V_i for the CV measurement. V_i is 1.0 V and 1.1 V vs. RHE in alkaline and acidic environments, respectively.

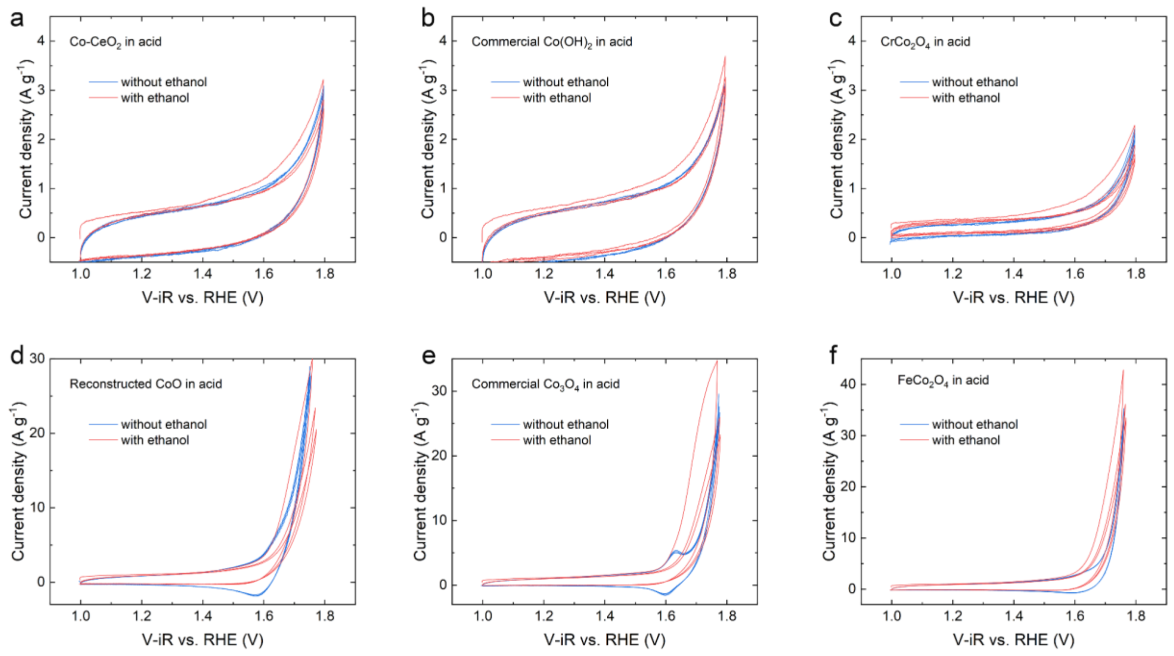

Supplementary Figure 20. Ethanol molecular was used as a chemical probe to detect OH* formation on the surface of the catalyst. The CVs (100 mV s^{-1}) with and without ethanol in the acidic electrolyte for the Co^{II} catalysts of (a) Co-CeO_2, (b) commercial $Co(OH)_2$ and (c) $CoCr_2O_4$, and the Co^{II}/Co^{III} catalysts of (d) reconstructed CoO, (e) commercial Co_3O_4 and (f) $FeCo_2O_4$.

Ethanol can react with OH*, one of the intermediates for OER. The formation of OH* is usually precedes the onset of OER, so the polarization curve in the electrolyte with ethanol should have an earlier onset. The similar CVs for all the three Co^{II} catalysts (Co-CeO_2, commercial $Co(OH)_2$ and $CoCr_2O_4$) show that OH* is difficult to form on their surface in an acidic environment, suggesting that the electron transfer step to form of Co^{III}-OH species may be turnover-limiting. For three other Co^{II}/Co^{III} catalysts, the onset is earlier with ethanol, even though the current decreases with cycles. This suggests the surface of the catalyst is poisoned by the carbon-containing species due to the strong binding with Co species, as evidenced by the absence of $Co^{III/IV}$ redox peaks from the CVs with ethanol[5].

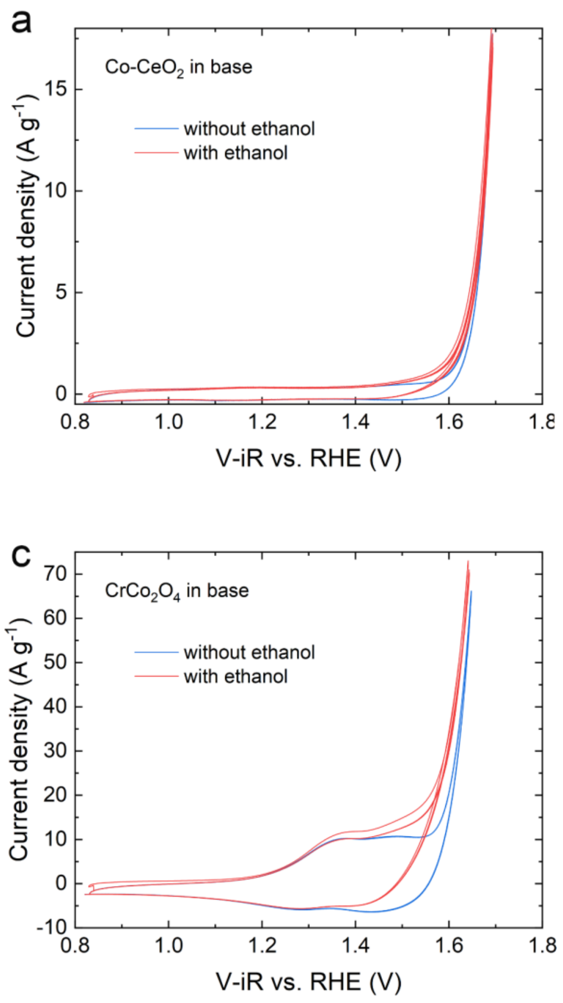

Supplementary Figure 21. Ethanol molecular was used as a chemical probe to detect OH* formation on the surface of the catalyst. The CVs (100 mV s^{-1}) with and without ethanol in an alkaline electrolyte for the Co^{II} catalysts of (a) $Co\text{-}CeO_2$, (b) commercial $Co(OH)_2$ and (c) $CoCr_2O_4$.

The polarization curves for the Co^{II} catalyst show an earlier onset with ethanol in an alkaline environment, different from that in an acidic environment. This suggests that the formation of OH* for these three catalysts is also easier in an alkaline environment. The unfavorable surface reconstruction to form the Co^{III}-OH* should be responsible for the poor OER activity in an acidic environment for the Co^{II} catalyst.

Supplementary Table 1. Summary of the Co branching ratio $I(L_3)/[I(L_3) + I(L_2)]$ for $Co_xMn_{1-x}O_y$ (x = 1, 0.9, 0.67, 0.5, 0.33 and 0.1)

	$I(L_3)$	$I(L_2)$	$I(L_3)/[I(L_3) + I(L_2)]$
x = 1	12.17	7.80	0.61
x = 0.9	11.45	7.37	0.61
x = 0.67	12.37	7.35	0.63
x = 0.5	11.76	6.74	0.64
x = 0.33	9.74	5.41	0.64
x = 0.1	6.83	3.06	0.69

Supplementary Table 2. Summary of the intensity ratio I(Co^{III})/I(Co^{II}) for $Co_xMn_{1-x}O_y$ (x = 1, 0.9, 0.67, 0.5, 0.33 and 0.1)

	I(CoII)	I(CoIII)	I(CoIII)/I(CoII)
x = 1	6.07	12.17	2.01
x = 0.9	7.43	11.45	1.54
x = 0.67	10.41	12.37	1.19
x = 0.5	10.87	11.76	1.08
x = 0.33	9.74	9.38	0.96
x = 0.1	6.83	5.04	0.74

Supplementary Table 3. Summary of the Co branching ratio $I(L_3)/[I(L_3) + I(L_2)]$ for the six representative samples

	$I(L_3)$	$I(L_2)$	$I(L_3)/[I(L_3) + I(L_2)]$
Co-CeO_2	13.13	5.83	0.69
Commercial $Co(OH)_2$	13.60	4.81	0.74
$CoCr_2O_4$	17.01	7.36	0.70
Reconstructed CoO	11.24	7.03	0.62
Commercial Co_3O_4	18.65	11.15	0.63
$FeCo_2O_4$	10.95	6.99	0.61

Supplementary Table 4. Summary of the intensity ratio I(Co^{III})/I(Co^{II}) for for the six representative samples

	I(Co^{II})	I(Co^{III})	I(Co^{III})/I(Co^{II})
Co-CeO_2	13.13	9.83	0.75
Commercial $Co(OH)_2$	13.60	6.44	0.47
$CoCr_2O_4$	17.01	10.59	0.62
Reconstructed CoO	7.57	11.24	1.49
Commercial Co_3O_4	8.32	18.65	2.24
$FeCo_2O_4$	6.11	10.95	1.79

Supplementary Table 5. Summary of the Co and Mn atomic percentages measured by XPS for $Co_xMn_{1-x}O_y$ (x = 0.9, 0.67, 0.5, 0.33 and 0.1)

	Co percentage measured by (atomic%)*	Mn percentage measured by (atomic%)*
x = 0.9	77.4	22.6
x = 0.67	55.5	44.5
x = 0.5	36.0	64.0
x = 0.33	38.2	61.8
x = 0.1	17.1	82.9

*: The Co and Mn atomic percentages were calculated with the peak area of the Co 2*p* and Mn 2*p*, respectively.

References in Supplementary Information

1. Huang J, *et al.* Modifying redox properties and local bonding of Co_3O_4 by CeO_2 enhances oxygen evolution catalysis in acid. *Nat. Commun.* **12**, 3036 (2021).

2. Qiao R, Chin T, Harris SJ, Yan S, Yang W. Spectroscopic fingerprints of valence and spin states in manganese oxides and fluorides. *Curr. Appl. Phys.* **13**, 544-548 (2013).

3. Gilbert B, *et al.* Multiple Scattering Calculations of Bonding and X-ray Absorption Spectroscopy of Manganese Oxides. *J. Phys. Chem. A* **107**, 2839-2847 (2003).

4. Binninger T, *et al.* Electrochemical Flow-Cell Setup for In Situ X-ray Investigations. *J. Electrochem. Soc.* **163**, H906-H912 (2016).

5. Zhang J, *et al.* Advances in Thermodynamic-Kinetic Model for Analyzing the Oxygen Evolution Reaction. *ACS Catal.* **10**, 8597-8610 (2020).
